# A Prologue of Practice

**Published:** 1910-09-03

**Authors:** Stephen Paget


### The Hospital

A JOURNAL OF

**The Medical Sciences and Hospital Administration.**

NEW SERIES. No. 186, VOL. VII. [No. 1258, VOL. XLVIII.] SATURDAY, SEPTEMBER 3 1910.

### EDUCATIONAL NUMBER.

### A PROLOGUE OF PRACTICE.

By STEPHEN PAGET.

SHAKESPEARE, for this or that of his plays, wrote a prologue: and some of our modern dramatists are not above following his example. A prologue, mostly, is spoken by some insignificant person, who takes no part in the play: some old actor, it may be, unable now to act Romeo or Hamlet, but familiar with all the traditions of the stage, and full of good advice to younger men, how to deserve and win the approval of the audience. Surely we ought to have a prologue to Practice, that drama of so many complex problems, and of such powerful human interest; and fair occasion for a prologue is afforded by the opening of another Hospital year.

This human interest, this wide and immediate sympathy, are among the chief influences which set a young man to enter our profession. Many of us fail, many have an uphill fight for a small income, and many die long before they reach the three score years and ten; but none of us need be dull, or, at the worst, if a dull time comes, it need not last. Always, if we wait, and look round, something turns up to occupy our thoughts and our wills, something worth doing or observing for its own sake. Everybody, of course, has times of dulness and of slackness, when nothing happens or seems likely to happen, and other men's lives look more pleasant, and other men's prospects more bright; but, in the long run, we doctors cannot complain that our lives are dull, save as all lives are now and again dull. Indeed, our work is so happily free from dulness that we suffer the more from that complaint, when at last we are too old for work.

That all of us are properly paid for our work, it would be too much to say, when we remember that the average of our earnings is less than £300 a year; but this dismal estimate includes those who are just starting in practice, or have retired from it, or hold, but do not use, a medical qualification. A man of good abilities, good character, and good health may be sure of making enough to support a wife and family. To make more than enough is, with most of us, to spend more than enough: and that is a great pleasure, to spend more than enough; but one may be content with enough. And it is certain that the public rates us, not by what we spend, but by what it thinks that we make; and has rather childish ideas of what we make, and how we make it.

To these two advantages of our work—that it is not dull, and that it brings us a decent livelihood—we must add this third advantage, that the public takes a favourable view of us. It calls us a noble profession; which is high praise from the public. We all of us like to be liked, and to be told, on Hospital Sunday, that we go about doing good. So we do: and that is another reason why we need not be dull so long as we have plenty of patients.

Still, it would be foolish to deny that some members of the public dislike us, and these unusual persons form an interesting group. Among them are they who talk of medical priestcraft, and of the tyranny of doctors, saying that our profession is a ring, a narrow and close trade union. They affirm that many of us are ignorant, conceited, avaricious, downright unscrupulous: that we experiment on hospital patients and perform unnecessary operations, for the hope of big fees or for the sheer delight of showing off: that we run after fads, go mad over vaccines and sera, and are neglectful of bedside observation: that we call everything appendicitis: that we are indifferent to the real hindrances in the way of national health, and are absurdly afraid of quite harmless or even beneficent germs. They look forward, some of them, to a Golden Age, within measurable distance, when vaccination will be abolished, and "sanitation" shall put an end to epidemic diseases: they proclaim the saving efficacy of exercise, fresh air, wholesome food, personal cleanliness, well-drained and well-ventilated houses, and a quiet mind; and they do not see the unkindness of thus proclaiming to the poor what fun it will be when there is no more poverty. They do believe, some of them, that the world, some day, will get well and keep well, by a sort of natural instinct, and as it were of itself, without "poisonous drugs," and without "the surgeon's knife." There will be no great need, in the Golden Age, of our services: we shall still be a fairly honest craft, to be employed in times of especial difficulty, but not more than that. Our pride, etiquette, pretensions, dogmatic beliefs, and whole explanation of the processes of the body will no longer be admitted or approved; and the world will breathe more freely once we are labelled as a trade and shorn of our present authority.

This way of thinking of us needs to be considered: for we are not the only profession that exercises authority: and, to a very notable extent, the dislike of doctors is part and parcel of a general dislike of all authority. Here is not the place to try to judge the merits and the demerits of rebellion against authority; it all depends on the intellectual and spiritual condition of each rebel. But, so far as our profession is concerned, we are ourselves the cause, more or less, why some people resent the dictatorial airs of medicine. They are tired, and no wonder, of hearing about our performances, our mighty works, our successful encounters with diseases. The names of Pasteur and Lister have tended of late years to throw into shade splendid achievements won in other fields of thought—in physics, engineering, literature, and art. Perhaps it is time that these two names should have a rest. Time is no object to them, for they are immortal.

By this revolt against authority, our's included, opportunity comes for the use of the weapons of prejudice and of sentiment. It would take too long here to estimate anti-vaccination and anti-vivisection, or to wonder why these two states of mind are so often found together, or to estimate the amount in the world to-day of that wilful credulity which accepts all sorts of malignant nonsense and is drawn by quacks as by a magnet. But our opponents have certain weapons of sentiment which are more honourable to them and deserve respectful consideration from us.

### The Simple Life.

First, we ought not to think lightly of the present fashionable sentiment in favour of a simpler rule of life. It runs into strange and extravagant ways, but a breath of open air goes where it runs. These fashionable ladies, who say that they mean to lead the simple life, are well aware that not every drug, nor every bit of medical advice, is worth taking: they know that the history of medicine and of surgery is full of systems, principles, theories, methods, discoveries, all slowly found wanting, and at last abandoned: and if they laugh at us, it does not follow that they are absolute fools. Till the world is too solemn, or, still worse, too clever, to laugh, there will always be room for a laugh at our expense; and we must not grudge the world its laughter, we who see so much of its pain. If this ill-used world, which has already swallowed such vast quantities of our tonics and our sedatives, should venture to try, instead of them, more air and exercise, more rest and sleep, less alcohol and meat, let us be thankful for that. We are, or ought to be, as indifferent as the world to mere trivial novelties of practice: the advertisement and the multiplication of them hardly help us in the day's work. To read what some of the newspapers say, one would imagine that sour milk could raise the dead, that every appendix ought to be removed in early childhood, and that a bottle of tonic is a bottle of Life. We shall be the gainers of good repute, not the losers, if we help to advance the cause of natural health. If, by more frugal diet, earlier hours, less excitement, and more rational dress, some of our patients can free themselves from our kind attentions, and go for years without us, shall we therefore be aggrieved?

### Sentiment and Science.

Next, we are bound to respect the present general desire to bring bodily affairs somehow into closer accord and line with spiritual affairs. Here is another sentiment, which may be called, without offence, fashionable, and runs into strange and extravagant ways; yet, wherever it goes, a breath of truth goes with it. We doctors see and despise the extravagances, but we must keep our eyes open to the sober facts of "spiritual healing." We know well, none better, how "Christian Science," with her impudent assertions, her false miracles, her ghastly meddling, has decoyed men, women, and children to death; and we find it hard to decide where "Christian Science" leaves off and "spiritual healing" begins. Yet we may be sure that some truth is mixed in the mass of untruth; and, if we lightly dismiss the whole subject from our minds, we shall be dismissing, along with its worthless fictions, its human facts. We call these facts by the convenient name of "suggestion"; yet our business is not to name but to understand and use them.

These two tendencies of thought, of desire, even of passionate desire, to simplify life, and to employ over the diseases of the body influences which may rightly be called spiritual—for one may as well talk Latin as Greek—are close to our work, and must be reckoned with, if we want to make every stroke of our work effective. They are influences of sentiment. Our influences are of science, not of sentiment. Here would be a deadlock, if the truth, the whole truth, and nothing but the truth, belonged either to our critics or to us. Happily for the world's fortunes, the truth does not behave in that mechanical way. And we shall do a good turn to the world if we preach our science and let the world, so far as it can, square the facts of science with the facts of sentiment.

### The Need for Interpreters.

Some of the very best of us are of opinion that a doctor's business is to act, not to talk, and that he ought to leave other people to do the talking; and it would be hard to have a higher standard of our work. Still, the very name of doctor means teacher; neither is it impracticable for a man or a woman in our profession to be a teacher, and that without self-advertisement. Besides, as it is now, our science has got overlaid with long Greek and Latin words, and the public thinks it hopelessly unintelligible. "As I have in many other things observed," says Lord Justice Fletcher Moulton, in his evidence before the Royal Commission on Vivisection, "the advance of science takes the workers in science more and more beyond the ken of the ordinary public, and their work grows to be a little understood, and much misunderstood, and I have felt that, as in many other cases, the need would come for interpreters between those who are carrying on scientific research and the public, in order to explain and justify their work."

The interpretation of our science must be guarded, lest it degenerate into popular descriptions of diseases and their remedies. Already, it may be, there is too much pathology talked over dinner-tables and in the Press. But a good occasion often comes for us doctors to do some teaching. We are the servants of the public: well, as Lord Sherbrooke said, we must educate our masters. Here is a new "hobby" for some of us, worthy of our vocation. If a doctor be minded to teach his neighbours, though it be no more than half-a-dozen lectures a year, he can do a lot of good. Our neighbours do want to hear what we have to tell them. Already a beginning has been made: in the teaching of the facts of tuberculosis, and in the teaching of rules for the care of infants. By such teaching many lives are saved, and no offence is given to anybody. We have no right, from fear of parading our art, to hide our science; it is our business, to get people to see the facts of disease. Without science, their sentiment is in danger of becoming extravagant, credulous, and false; with science, their sentiment will increase the happiness of a world which is not in any danger of being too happy.

